# Clinicopathological, Molecular, and Economic Outcomes of Bethesda III and IV Thyroid Nodules Following Implementation of a Publicly Funded ThyroSeq v3 Program: A Retrospective Two-Center Cohort Study

**DOI:** 10.3390/cancers18162695

**Published:** 2026-08-20

**Authors:** Gianluca Savoia, Sarah B. Payne, Matthew Vaitzner, Saruchi Bandargal, Michael P. Hier, Marco A. Mascarella, Keith Richardson, Alex Mlynarek, Nader Sadeghi, Sabrina Daniela Silva Wurzba, Rawan Azzam, Elliot J. Mitmaker, Brian M. Gilfix, Marc Pusztaszeri, Véronique-Isabelle Forest, Richard J. Payne

**Affiliations:** 1 Faculty of Medicine and Health Sciences, McGill University, Montreal, QC H3A 2B4, Canada; 2 Lower Canada College, Montreal, QC H4A 2L1, Canada; 3 Faculty of Education, Department of Kinesiology and Physical Education, McGill University, Montreal, QC H3A 2B4, Canada; matthew.vaitzner@mail.mcgill.ca; 4 Department of Otolaryngology-Head and Neck Surgery, University of Ottawa, Ottawa, ON K1N 6N5, Canada; 5 Department of Otolaryngology-Head and Neck Surgery, McGill University, Jewish General Hospital, Montreal, QC H3T 1E2, Canada; 6 Department of Otolaryngology-Head and Neck Surgery, McGill University, Royal Victoria Hospital, Montreal, QC H4A 3J1, Canadarawan.azzam@mail.mcgill.ca (R.A.); 7 Division of General Surgery, Department of Surgery, McGill University Health Center, Montreal, QC H4A 0B1, Canada; elliot.mitmaker@mcgill.ca; 8 Division of Medical Biochemistry, Department of Specialized Medicine, McGill University Health Centre, Montreal, QC H4A 0B1, Canada; brian.gilfix@mcgill.ca; 9 Department of Pathology, Jewish General Hospital, McGill University, Montreal, QC H3A 2B4, Canada

**Keywords:** indeterminate thyroid nodules, ThyroSeq v3, Bethesda III, Bethesda IV, molecular diagnostics

## Abstract

**Background/Objectives**: Since 2021, indeterminate thyroid nodules have been routinely assessed using molecular testing through Quebec’s provincial ThyroSeq v3 pilot project. The primary objective of our study was to compare the molecular and clinicopathological characteristics of Bethesda III and Bethesda IV thyroid nodules evaluated through the pilot project. The secondary objective was to assess the program’s economic impact. **Methods**: This retrospective study included 934 patients (mean age 55.7 ± 14.3 years; 79.3% female; mean nodule size 2.2 ± 0.8 cm) from two McGill University teaching hospitals in Quebec, comprising 593 Bethesda III and 341 Bethesda IV nodules that underwent molecular testing between 2021 and 2025. Eligible nodules were TI-RADS 3 or 4, measured 1–4 cm, and Bethesda III nodules required two consecutive cytology results before testing. Clinicopathological characteristics, histopathological diagnoses, molecular findings, and mutational profiles were analyzed. A projected 10-year cost analysis was also performed. **Results**: Of the 934 nodules tested, 280 demonstrated a genetic alteration. The risk of malignancy among surgically resected ThyroSeq v3-positive Bethesda III and IV nodules was 68% and 78%, respectively. Compared with Bethesda III nodules, Bethesda IV nodules demonstrated significantly greater vascular invasion (*p* = 0.029) and prevalence of oncocytic carcinomas (*p* = 0.010). Molecular testing was associated with an estimated reduction of 1.49 million Canadian dollars in healthcare costs over 10 years. **Conclusions**: Although Bethesda IV nodules exhibited more oncocytic carcinomas and invasion, molecular mutation class distribution and the prevalence of aggressive pathological features were similar between Bethesda categories. Together with the demonstrated economic savings, these findings support continued implementation of publicly funded ThyroSeq v3 testing to improve management of indeterminate thyroid nodules while reducing unnecessary surgery.

## Simple Summary

Thyroid nodules are common, but some cannot be clearly identified as benign or malignant after biopsy. Traditionally, these indeterminate nodules have been managed through observation or surgery. In Quebec, a publicly funded pilot project introduced ThyroSeq v3 molecular testing to guide treatment for selected patients with indeterminate thyroid nodules. This study compares the molecular, pathological, and clinical features of the two main types of indeterminate nodules and evaluates the economic impact of the program. Although one group had more oncocytic malignancies and vascular invasion, the overall molecular profiles were similar, indicating no distinguishing patterns between groups on biopsy and emphasizing the depth provided by molecular testing. We found that molecular testing reduces unnecessary surgeries and may offer long-term cost savings, supporting its continued use to improve management and optimize healthcare resources.

## 1. Introduction

Thyroid nodules are a common endocrine disorder, found in up to 60% of adults [1]. Ultrasound-guided fine-needle aspiration (USFNA) is the gold-standard tool for cytological assessment of these thyroid nodules, interpreted using the standardized Bethesda System for Reporting Thyroid Cytopathology (TBSRTC) [2,3]. Despite its widespread usage, roughly 20–30% of biopsied thyroid nodules end up being classified as cytologically indeterminate, most often falling into the Bethesda III (atypia of undetermined significance; AUS) and Bethesda IV (follicular neoplasm; FN) categories [4]. These nodules pose a significant diagnostic challenge because cytology alone cannot reliably distinguish benign from malignant lesions, creating uncertainty regarding appropriate clinical management [5]. Consequently, diagnostic surgery has historically been recommended for many patients, despite the fact that a large proportion of these nodules are ultimately shown to be benign on surgical pathology [6,7].

In the past two decades, management recommendations for indeterminate thyroid nodules (ITNs) have greatly evolved. The first iteration of TBSRTC in 2009 recommended repeat USFNA as the preferred approach for Bethesda III nodules and diagnostic lobectomy for Bethesda IV nodules [8]. The 2017 and 2023 updates of TBSRTC extended the use of diagnostic lobectomy to Bethesda III nodules and introduced molecular testing as a management strategy for both Bethesda III and IV, based on the 2015 American Thyroid Association (ATA) guidelines addressing thyroid nodules [3,5,9]. Nevertheless, all iterations of TBSRTC have emphasized that management should not rely on cytological classification alone but also integrate clinical, sonographic, molecular and patient-specific factors into treatment strategy decision making [3,8,9].

Molecular testing has therefore emerged as an important tool in helping guide the management of ITNs [10]. In 2021, the Quebec Ministry of Health launched a publicly funded province-wide pilot program evaluating ThyroSeq v3 for patients with Bethesda III and IV nodules measuring 1–4 cm in size and classified as TI-RADS 3 or 4 according to sonographic evaluation [4]. The ThyroSeq v3 molecular test is an NGS-based analysis of SNVs/indels, gene fusions, gene expression alterations, and copy number alterations in targeted regions of 112 thyroid-cancer-related genes [11]. The test reports results as positive for a disease-associated alteration and gives the probability of cancer. The initial evaluation of this program suggested that molecular testing substantially reduced unnecessary surgery while maintaining cost-effectiveness within a publicly funded healthcare system [4,12].

In 2022, the World Health Organization (WHO) Classification of Thyroid Neoplasms brought forward a framework that suggested classic papillary thyroid carcinoma (PTC) is predominantly characterized by *BRAF*-associated alterations. This includes isolated *BRAF V600E* or *BRAF V600E*-like alterations (e.g., *RET*/*PTC*, *BRAF* fusions, and *BRAF V600E*-like gene expression alterations). Follicular derived tumors such as follicular thyroid carcinoma (FTC) and invasive encapsulated follicular variant of papillary thyroid carcinoma (IEFVPTC) are predominantly characterized by *RAS*-associated alterations. This includes *RAS* pathogenic variants or *RAS*-like alterations (e.g., *BRAF K601E* mutation, *THADA* fusion, *RAS*-like gene expression alterations). This molecular classification reflects distinct biological pathways of thyroid tumorigenesis which could potentially complement traditional histopathological classifications [13,14].

Although Bethesda III and Bethesda IV nodules both fall into the ITN category, they differ not only in estimated risk of malignancy (ROM) and management recommendations but also in their underlying tumorigenic biology. Therefore, evaluating Bethesda III and IV nodules within our large, real-world molecular testing program may provide additional insight into the underlying biological characteristics and help identify potential avenues for optimizing the clinical application of molecular testing.

The primary objective of this study was therefore to compare the molecular, histopathological, and clinical characteristics of Bethesda III and Bethesda IV nodules evaluated within the Quebec pilot ThyroSeq v3 testing program. A secondary objective was to evaluate the economic impact of this province-wide molecular testing strategy four years after its implementation.

## 2. Materials and Methods

### 2.1. Study Design

This retrospective chart review encompassed 934 patients from two teaching hospitals affiliated with McGill University in Quebec. The study included 593 Bethesda III nodules and 341 Bethesda IV nodules, all assessed as part of a provincial pilot project. Researchers collected comprehensive data on patient demographics, preoperative USFNA results, molecular testing, and postoperative pathology. Molecular analyses were conducted at the Molecular & Genomic Pathology Lab within the Pittsburgh University Medical Laboratory in Pennsylvania, United States. Cost assessments were based on information provided by the Centres for Medicare and Medicaid Services, as well as the Institut national d’excellence en santé et en services sociaux (INESSS), using provincial remuneration values derived from the Régie de l’assurance maladie du Québec RAMQ Manuel des spécialistes [4,15]. All costing is in Canadian dollars.

### 2.2. Study Sample

The medical records of patients aged 18 years or older with Bethesda III or IV thyroid nodules who underwent molecular testing through the provincial ThyroSeq v3 pilot project between November 2021 and November 2025 were reviewed. Inclusion criteria required nodules to measure between 1 and 4 cm, be classified as intermediate suspicion on ultrasound (TI-RADS 3 or 4), and have Bethesda III or IV cytology. Bethesda IV nodules required a single cytopathology result, whereas Bethesda III nodules required two consecutive Bethesda III cytology results before molecular testing. These eligibility criteria were designed to enrich the cohort for patients in whom molecular testing would meaningfully influence clinical decision-making by excluding nodules at high risk of malignancy that would generally proceed directly to surgery (e.g., TI-RADS 5, Bethesda V or VI, or nodules > 4 cm), as well as nodules at low risk of malignancy that would typically undergo surveillance (e.g., TI-RADS 1–2, Bethesda II, or a single Bethesda III cytology result).

### 2.3. Data Collection

Data collected included age, sex, Bethesda score, ThyroSeq v3 results (positive, negative, or currently negative with cancer probability if positive), variant allele frequency, copy number alteration, and gene expression profile. Nodule location, size, and atypia were recorded from digital reports. Final surgical histopathology was reviewed for malignancy status, subtype/variant, size, tumor capsule invasion, lymphovascular invasion, vascular invasion, extrathyroidal extension, and lymph node metastasis. Non-invasive follicular thyroid neoplasm with papillary-like nuclear features (NIFTP) on final pathology was grouped with malignant outcomes given that it requires surgical excision. [14,16,17]. For vascular invasion, minimally invasive was defined as capsular invasion only, without vascular invasion. Aggressive carcinoma was defined by the presence of gross extrathyroidal extension (ETE), lymphovascular invasion (LVI), lymph node metastasis (LNM), high-risk histological features, or aggressive histological subtypes (including tall cell, columnar, hobnail/micropapillary, and diffuse sclerosing variants) [18]. Invasive included tumors with focal (<4 foci) or extensive (≥4 foci) vascular invasion, as well as widely invasive carcinomas [19]. A cost analysis was conducted using a 10-year projection, incorporating the costs of ThyroSeq v3, provincial estimates for surgical management, post-operative hypothyroidism treatment, and ongoing surveillance (Table 7) [4,15].

### 2.4. Statistical Analysis

Welch’s two-sample t-test compared continuous variables, summarized by mean and standard deviation, between Bethesda III and IV groups. Pearson’s chi-squared test analyzed categorical variables presented as frequencies and percentages. Fisher’s exact test was used for 2 × 2 contingency tables, while the Monte Carlo Fisher-Freeman-Halton exact test addressed sparse multi-category tables. Analysis of malignancy risk was limited to surgically resected nodules with available final pathology. Two-sided *p* values and effect estimates (risk differences, risk ratios, or odds ratios with 95% confidence intervals) are reported. Statistical analyses were conducted using IBM SPSS Statistics Version 29.0 (IBM Corp., Armonk, NY, USA).

## 3. Results

Table 1 summarizes the baseline patient and nodule characteristics. The overall mean age was 55.7 ± 14.3 years, and age and nodule location were similarly distributed between Bethesda III and Bethesda IV nodules. Bethesda III nodules occurred more frequently in female patients (81.8% vs. 75.1%, *p* = 0.015), while Bethesda IV nodules were slightly larger (2.3 ± 0.8 cm vs. 2.2 ± 0.8 cm, *p* = 0.009). A significantly greater proportion of Bethesda IV nodules were positive on ThyroSeq v3 compared to Bethesda III nodules (41.9% vs. 23.1%, *p* < 0.001), and consequently, a higher proportion of Bethesda IV patients underwent surgical management (29.6% vs. 17.2%, *p* < 0.001).

Among nodules with available surgical pathology, the observed malignancy rate for ThyroSeq v3 positive surgically resected thyroid nodules was 67.6% in Bethesda III nodules and 78.2% in Bethesda IV nodules (Table 2).

Table 3 shows the 277 nodules with available molecular classification eligible to be grouped as *BRAF*-like, *RAS*-like and non-*BRAF*/non-*RAS* (NBNR) [14,20]. The distribution of molecular classes did not differ significantly between B3 and B4 nodules (*p* = 0.540). *RAS*-like alterations were the most common molecular subtype in both groups (68.5% vs. 65.1%), followed by NBNR alterations (28.0% vs. 33.0%), while *BRAF*-like alterations were uncommon (3.6% vs. 1.8%).

In the 203 nodules with available surgical pathology, the distribution of histopathologic diagnoses according to the WHO classification differed significantly between Bethesda III and Bethesda IV nodules (*p* = 0.010) (Table 4). Bethesda III nodules more frequently demonstrated benign lesions such as follicular adenoma and nodular disease, whereas Bethesda IV nodules demonstrated a greater proportion of oncocytic follicular variant of papillary thyroid carcinoma (OFVPTC) and oncocytic carcinoma (OCA). Despite these differences, the proportion of tumors classified as aggressive (Table 5) did not differ significantly between Bethesda III and Bethesda IV nodules (11.8% vs. 14.9%, *p* = 0.517).

Among 73 nodules with available vascular invasion data (Table 6), invasive carcinoma occurred significantly more frequently in Bethesda IV nodules than in Bethesda III nodules (21.1% vs. 2.9%; Fisher’s exact test, *p* = 0.029).

Of the 934 pilot project patients included in the study, 280 had positive molecular test results necessitating surgical intervention, while 654 had negative results and were managed with ultrasound surveillance. Integrating ThyroSeq v3 into the 10-year management plan for this patient cohort yielded an estimated total cost of CAD 8,688,880.40. Conversely, if ThyroSeq v3 was not utilized and all patients underwent diagnostic surgery, the projected 10-year total cost would rise to CAD 10,175,930.00 (Table 7).

Stratifying the analysis by Bethesda category revealed that the greatest economic benefit occurred with Bethesda III nodules (Table 8). Molecular testing reduced the estimated 10-year management cost of Bethesda III nodules from CAD 6,460,735.00 to CAD 5,145,265.60, resulting in net savings of CAD 1,315,469.40. Savings for Bethesda IV nodules were more modest, with a reduction in CAD 171,580.20 from CAD $3,715,195 to CAD 3,543,615.00 (Table 9).

**Table 7:** Cost estimates (in Canadian dollars).

Cost Category	Cost (CAD)
Assumed cost of ThyroSeq v3 in Quebec [15]	$4785.00
Cost of thyroidectomy in Quebec [4]	$9314.30
Cost of managing thyroidectomy hypothyroidism/patient/year in Quebec [4]	$158.07
Cost of surveillance of ITN/patient/year (ultrasound + clinic visit) in Quebec [4]	$178.76

Costs were derived from the Centres for Medicare and Medicaide Services, as well as the Institut national d’excellence en santé et en services sociaux (INESSS) based on provincial remuneration values from RAMQ Manuel des spécialistes.

**Table 8:** Cost savings breakdown (in Canadian dollars)—Bethesda III.

Scenario	Cost Components	Cost (CAD)
ThyroSeq v3 management over 10 years in Bethesda III (*n* = 593)	ThyroSeq v3 testing (593 nodules)	$2,837,505.00
	Total cost associated with surgery (137)	$1,492,615.00
	Surgical management (137 patients)	$1,276,059.10
	Post-thyroidectomy hypothyroidism management (137 patients over 10 years)	$216,555.90
	Surveillance of nodules managed non-operatively (456 nodules, 10 years)	$815,145.60
	Total cost with ThyroSeq v3	$5,145,265.60
Standard management assuming ThyroSeq v3 not used in Bethesda III (*n* = 593)	Surgical management (593 nodules)	$5,523,379.90
	Post-thyroidectomy hypothyroidism management (593 nodules, 10 years)	$937,355.10
	Total cost without ThyroSeq v3	$6,460,735.00
Cost savings	Net cost savings over 10 years	$1,315,469.40

Costs represent projected expenditures over a 10-year time horizon. Surgical costs include thyroidectomy and long-term postoperative hypothyroidism management. Surveillance costs include annual ultrasonography and outpatient follow-up.

**Table 9:** Cost savings breakdown (in Canadian dollars)—Bethesda IV.

Scenario	Cost Components	Cost (CAD)
ThyroSeq v3 management over 10 years in Bethesda IV (*n* = 341)	ThyroSeq v3 testing (341 nodules)	$1,631,685.00
	Total cost associated with surgery (143)	$1,557,985.00
	Surgical management (143 patients)	$1,331,944.90
	Post-thyroidectomy hypothyroidism management (143 patients over 10 years)	$226,040.10
	Surveillance of nodules managed non-operatively (198 patients, 10 years)	$353,944.80
	Total cost with ThyroSeq v3	$3,543,614.80
Standard management assuming ThyroSeq v3 not used in Bethesda IV (*n* = 341)	Surgical management (341 nodules)	$3,176,176.30
	Post-thyroidectomy hypothyroidism management (341 nodules, 10 years)	$539,018.70
	Total cost without ThyroSeq v3	$3,715,195.00
Cost savings	Net cost savings over 10 years	$171,580.20

Costs represent projected expenditures over a 10-year time horizon. Surgical costs include thyroidectomy and long-term postoperative hypothyroidism management. Surveillance costs include annual ultrasonography and outpatient follow-up.

## 4. Discussion

This study represents one of the largest evaluations of the molecular, clinicopathological, and economic features of Bethesda III and IV ITNs using a publicly funded molecular testing program. While Bethesda IV nodules showed more oncocytic carcinomas, and greater vascular invasion, both ITN classes had similar distributions of molecular mutation classes and aggressive pathologies. Additionally, our analysis indicates that molecular testing remains economically favorable in a publicly funded system, highlighting both the economic and clinical benefits of molecular testing for thyroid nodules in a publicly funded healthcare system.

The ROM reported in our study was 67.6% and 78.2% for Bethesda III and IV respectively, exceeding the expected mean ROM reported in the latest edition of TBSRTC 2023 which lists the mean ROM as 22% and 30% respectively [3]. This discrepancy was expected and reflects a selection bias as our cohort consisted of ThyroSeq v3 positive nodules therefore enriching our study population for malignancy. This finding highlights molecular testing’s ability to accurately differentiate malignant from non-malignant nodules highlighting its role in guiding surgical decision making. Considering that our observed ROM effectively reflects the positive predictive value (PPV) of ThyroSeq v3, our observed PPVs of 67.6% and 78.2% for Bethesda III and IV respectively were slightly higher than those previously reported by Desai et al. (61.7% and 71.0%) and Nikiforov et al. (64.0% and 68.0%) further supporting the clinical utility of ThyroSeq v3 across multiple clinical settings [14,21]. The discrepancy may, in part, reflect the more selective inclusion criteria used in our study. Unlike previous ThyroSeq v3 validation studies, which included all eligible Bethesda III and IV nodules, our cohort was restricted to intermediate-risk nodules (TI-RADS 3–4, 1–4 cm), with Bethesda III nodules additionally requiring two consecutive Bethesda III cytology results prior to molecular testing. Patients with nodules at high risk of malignancy that would generally proceed directly to surgery (e.g., TI-RADS 5 or nodules > 4 cm), as well as those at low risk that would typically undergo surveillance (e.g., TI-RADS 1–2 or a single Bethesda III cytology result), were excluded. Consequently, our cohort more closely represents the clinical population in whom molecular testing is intended to influence management and, therefore, those who would otherwise be the most likely candidates for diagnostic surgery.

Despite no significant differences in the distribution of molecular classes (*BRAF*-like, *RAS*-like, and NBNR) between Bethesda III and IV nodules, important histopathologic differences were observed. Bethesda IV nodules demonstrated a greater proportion of oncocytic neoplasms, whereas Bethesda III contained a higher proportion of benign follicular pathologies such as follicular adenomas and thyroid follicular nodular disease. Interestingly, mirroring the similar distribution of *RAS*-like mutations across the two Bethesda categories was the comparable frequency of invasive encapsulated papillary thyroid carcinomas (IEFVPTC). These findings reflect the TBSRTC 2023 description of the Bethesda IV category partially composed of lesions with oncocytic differentiation such as OFVPTC and OCA which were concentrated in the Bethesda IV category in our cohort [3].

In addition to the histopathological differences between Bethesda III and IV, was the enrichment of invasive carcinoma in Bethesda IV [22]. This finding is biologically plausible as follicular-derived carcinoma characteristically spread through vascular invasion contrary to papillary thyroid carcinomas which prefer to spread via the lymphatic route [13,23,24]. Nevertheless, despite these histopathologic differences, the similar distribution of *RAS*-like molecular alterations, infiltrative encapsulated follicular variant papillary thyroid carcinomas (IEFVPTCs), and classical papillary thyroid carcinomas (PTCs) between Bethesda III and Bethesda IV nodules suggests that cytologic classification alone does not fully capture the underlying biological heterogeneity of indeterminate thyroid nodules (ITNs). Although the Bethesda System remains invaluable for risk stratification and clinical management, it is based primarily on morphologic features and therefore cannot reliably distinguish tumors with distinct molecular pathogenesis but overlapping cytologic appearances [3]. In this context, our findings underscore the complementary role of ThyroSeq v3, which provides biologically relevant molecular characterization beyond cytomorphology. By identifying the genomic alterations driving morphologically similar ITNs, ThyroSeq v3 refines risk assessment, offers insights into the likely histopathologic diagnosis, and supports more individualized clinical decision-making.

Providing more individualized decision-making is essential for determining the optimal extent of surgery, as the surgical scope must balance oncologic benefit with postoperative morbidity. Total thyroidectomy (TT), compared with hemithyroidectomy (HT), has been associated with a higher risk of transient hypocalcemia and hypoparathyroidism, reflecting the greater risk of parathyroid injury associated with a more extensive operation. Similarly, TT carries a higher risk of temporary vocal fold paralysis than HT [25]. These findings further emphasize the importance of accurate preoperative risk stratification to guide the extent of surgery while minimizing treatment-related morbidity. Surgical management should be tailored to the specific molecular alteration identified rather than treating all positive results uniformly. In general, *RAS*-like alterations may be appropriately managed with hemithyroidectomy in otherwise low-risk patients, whereas *BRAF*-like alterations may support consideration of a more extensive surgical approach because of their substantially higher probability of malignancy [26,27]. Ultimately, management should integrate molecular findings with clinical, sonographic, and patient-specific risk factors. Incidental thyroid carcinomas can also be detected postoperatively in various Bethesda categories, with a reported frequency of 9% in Bethesda II nodules, and 17.5% and 19% in Bethesda III and IV nodules, respectively [28]. This observation highlights the higher underlying malignancy potential of ITNs, particularly Bethesda III and IV nodules, and underscores the importance of precise risk stratification made possible by molecular testing.

Beyond the molecular and pathological findings, this study also provides further support for the continued implementation of Quebec’s publicly funded ThyroSeq v3 program. Using similar cost assumptions, calculations, and economic models as the initial 2-year post-implementation province-wide evaluation, we found the publicly funded ThyroSeq v3 pilot project to remain cost-effective while continuing to provide clinically valuable stratification of ITNs [4]. Over a projected ten-year span, molecular testing was estimated to save about CAD 1.49 million within this group. Although the test seemed cost-effective for both Bethesda III and IV nodules, the largest financial savings were seen among Bethesda III patients. This is likely because these cases have a lower chance of malignancy before testing compared to Bethesda IV, making it easier to avoid unnecessary diagnostic surgeries, as seemed to be the case in our cohort [5,29].

In addition to the financial advantages of implementing province-wide molecular testing, there are notable direct gains from using operating rooms (OR) more efficiently. For instance, 654 patients in our cohort with negative ThyroSeq v3 results avoided diagnostic surgery. Assuming a typical hemi-thyroidectomy, including anesthesia and perioperative time, takes roughly two hours, this corresponds to approximately 1308 h of OR time saved, which equals 163 days over ten years, or about 16 OR days per year. In our publicly funded healthcare system, where OR access is limited, reclaiming this time could allow for many additional procedures. Furthermore, avoiding diagnostic surgery may play an essential role in preserving patients’ health-related quality of life. A recent scoping review reported a modest improvement in quality of life (QOL) for patients who underwent molecular testing for ITNs compared to surgery, and also noted a significant enhancement in QOL among individuals who ultimately received surgery following a suspicious or positive result [30]. Combined with the molecular and pathological findings presented in this study, these outcomes further reinforce the importance of ongoing public funding of ThyroSeq v3 in Quebec, as the program demonstrates economic sustainability, optimizes operating room usage, and advances patient-centered care through more precise management of ITNs.

Lastly, beyond potentially reducing unnecessary surgeries, molecular testing may help identify patients who could be candidates for alternative non-surgical therapies, such as thermal ablation. Techniques such as radiofrequency and microwave ablation are becoming increasingly popular for treating benign lesions, and ongoing research is exploring their potential use for certain malignant nodules [31,32,33]. Molecular testing can support more individualized patient management by identifying those who might benefit from less invasive, non-surgical procedures such as thermal ablation.

This study has several limitations. First, its retrospective design is inherently subject to selection and information biases; for example, potentially relevant baseline characteristics, such as BMI and smoking status, were not routinely collected in the database and therefore could not be evaluated. Second, histopathological analysis was limited to surgically resected nodules. Consequently, Bethesda III (AUS) nodules managed non-operatively lacked pathological confirmation, introducing verification (selection) bias, as patients who proceeded to surgery were more likely to have positive molecular findings or other concerning clinical features. Although 280 nodules tested positive by ThyroSeq v3, histopathological data were available for only 203 cases. This incomplete follow-up may reflect delays between molecular testing and surgery, surgery performed outside our two McGill University healthcare centers, or pathology reports that were unavailable at the time of data collection. The cost analysis required several assumptions. For example, postoperative hypothyroidism following lobectomy was assumed, while the costs associated with postoperative complications and quality-adjusted life years were not incorporated into the economic model. However, the assumptions, inclusion criteria, and costing methodology, aside from the updated ThyroSeq v3 cost, were intentionally kept identical to those used in the previously published evaluation of the Quebec ThyroSeq v3 pilot project by Lévesque et al., allowing direct comparison between the two analyses [4]. Finally, we acknowledge that future multicenter validation and external assessment will be necessary to determine the generalizability of our findings from these two institutions.

## 5. Conclusions

In summary, our cohort study of 934 participants who underwent molecular testing as part of the publicly funded Quebec ThyroSeq v3 pilot project suggested both favorable clinical and economic implications. Bethesda IV nodules tended to be oncocytic with a higher proportion of invasion; however, molecular class distribution and aggressive features were comparable between both classes of ITNs, highlighting the clinical utility of ThyroSeq v3 in refining cytological ambiguity and guiding management decisions. Furthermore, economic analysis indicates that, over a ten-year period, molecular testing is financially advantageous for both Bethesda III and IV nodules.

## Figures and Tables

**Table 1:** Patient and nodule characteristics.

Characteristic	Overall (*n* = 934)	Bethesda III (*n* = 593)	Bethesda IV (*n* = 341)	*p* Value
Age, years, mean ± SD	55.7 ± 14.3	56.1 ± 14.1	55.1 ± 14.7	0.314 ^a^
Female sex, *n* (%)	741 (79.3)	485 (81.8)	256 (75.1)	0.015 ^b^
Nodule size, cm, mean ± SD	2.2 ± 0.8	2.2 ± 0.8	2.3 ± 0.8	0.009 ^a^
Nodule location				0.204 ^b^
Right lobe	503 (53.9)	325 (54.8)	178 (52.2)	
Left lobe	374 (40.0)	238 (40.1)	136 (39.9)	
Isthmus	57 (6.1)	30 (5.1)	27 (7.9)	
Positive ThyroSeq v3 result, *n* (%)	280 (30.0)	137 (23.1)	143 (41.9)	<0.001 ^b^
Underwent surgery, *n* (%)	203 (21.7)	102 (17.2)	101 (29.6)	<0.001 ^b^

Values are presented as mean ± standard deviation (SD) or number (%), as appropriate. ^a^ Welch *t*-test. ^b^ Pearson chi-square.

**Table 2:** Risk of malignancy, with NIFTP among surgically resected ThyroSeq v3 nodules.

Outcome	Bethesda III, *n* (%)	Bethesda IV, *n* (%)	*p* Value	Effect (BIV vs. BIII)	95% CI
Non-malignant	33 (32.4)	22 (21.8)			
Malignant (incl. NIFTP)	69 (67.6)	79 (78.2)	0.090	Risk difference: 10.6 pp	−1.7 to 22.4 pp
				Risk ratio: 1.16	0.98 to 1.37
				Odds ratio: 1.72	0.91 to 3.18

Risk of malignancy was calculated among surgically resected ThyroSeq v3-positive nodules. NIFTP was grouped with malignant outcomes. NIFTP, non-invasive follicular thyroid neoplasm with papillary-like nuclear features.

**Table 3:** Comparison of molecular driver class distribution between Bethesda III and Bethesda IV thyroid nodules.

Molecular Class	Bethesda III (%)	Bethesda IV (%)	Global *p* Value
*RAS*-like	115 (68.5)	71 (65.1)	0.540
*BRAF*-like	6 (3.6)	2 (1.8)	
Non-*BRAF*-non-*RAS*	47 (28.0)	36 (33.0)	

Molecular alterations were grouped into *BRAF*-like, *RAS*-like, and non-*BRAF*/non-*RAS* (NBNR) molecular classes according to previously published molecular classifications [14,20].

**Table 4:** Comparison of WHO histopathological diagnoses between Bethesda III and Bethesda IV thyroid nodules.

Histopathology	Bethesda III (%)	Bethesda IV (%)	Global *p* Value
			0.010
IEFVPTC	26 (25.5)	21 (20.8)	
*BRAF K601E*	1	0	
*DICER1*	0	1	
*HRAS*, *EIF1AX*	1	0	
*HRAS*	4	3	
*KRAS*	5	1	
*NRAS*	10	9	
*PTEN*	0	1	
*THADA*/*IGF2BP3*	3	2	
*PAX8*/*PPARG*	1	0	
*CNA*	1	4	
PTC	23 (22.5)	18 (17.8)	
*BRAF V600E*	4	2	
*EIF1AX*	1	0	
*HRAS*	2	5	
*KRAS*	2	0	
*NRAS*	8	4	
*NRAS*, *VHL*	1	0	
*PTEN*	1	0	
*TERT*	0	1	
*THADA*/*IGF2BP3*	0	2	
*PAX8*/*PPARG*	0	1	
*CCDC6*/*RET*	1	0	
*NTRK3*/*ETV6*	1	1	
*EML4*/*ALK*	1	0	
*CNA*	0	2	
*GEP*	1	0	
NIFTP	12 (11.8)	18 (17.8)	
*BRAF K601E*	0	1	
*HRAS*	2	3	
*KRAS*	1	2	
*KRAS*, *EIF1AX*	0	1	
*NRAS*	6	3	
*NRAS*, *TSHR*	0	1	
*THADA*/*IGF2BP3*	1	5	
*PAX8*/*PPARG*	0	1	
*CNA*	1	1	
*GEP*	1	0	
Follicular Adenoma	17 (16.7)	10 (9.9)	
*BRAF V600E*	1	0	
*BRAF K601E*	0	2	
*DICER1*	1	0	
*EIF1AX*	0	1	
*HRAS*	1	2	
*KRAS*	2	0	
*KRAS*, *EIF1AX*	0	1	
*NRAS*	4	2	
*PIK3CA*	1	0	
*TSHR*	2	0	
*TP53*	1	0	
*THADA*/*IGF2BP3*	1	0	
*PAX8*/*PPARG*	1	0	
*CNA*	2	2	
FTC	6 (5.9)	3 (3.0)	
*NRAS*	1	0	
*NRAS*, *TERT*	1	0	
*HRAS*, *TERT*	1	0	
*THADA*/*IGF2BP3*	1	1	
*PAX8*/*PPARG*	1	2	
*CNA*	1	0	
OFVPTC	0 (0)	7 (6.9)	
*EIF1AX*	0	2	
*KRAS*	0	1	
*TP53*, *TP53*, *TP53*	0	1	
*TERT*	0	1	
*CNA*	0	2	
OCA	1 (1.0)	10 (9.9)	
*EIF1AX*	1	0	
*KRAS*	0	1	
*EML4*/*ALK*	0	1	
*CNA*	0	8	
Oncocytic Adenoma	5 (4.9)	6 (5.9)	
*EIF1AX*	1	0	
*KRAS*	1	1	
*NRAS*	1	0	
*CNA*	2	4	
*GEP*	0	1	
Follicular Nodular Disease	9 (8.8)	4 (4.0)	
*BRAF V600S*	1	0	
*BRAF K601E*	0	1	
*EIF1AX*	1	0	
*EIF1AX*, *PIK3CA*	1	0	
*KRAS*	1	0	
*NRAS*	4	0	
*PTEN*	0	1	
*CNA*	1	0	
*GEP*	0	2	
WDT-UMP	1 (1.0)	1 (1.0)	
*HRAS*	0	1	
*KRAS*	1	0	
PDTC	1 (1.0)	2 (2.0)	
*EIF1AX*, *GNAS*, *TERT*	0	1	
*PAX8*/*PPARG*	1	0	
*GEP*	0	1	
Benign (Other)	1 (1.0)	1 (1.0)	
*HRAS*, *DICER1*	1	0	
*EIF1AX*	0	1	

Histopathological diagnoses were classified according to the 2022 World Health Organization (WHO) Classification of Thyroid Neoplasms [13]. IEFVPTC, invasive encapsulated follicular variant papillary thyroid carcinoma; PTC, papillary thyroid carcinoma; NIFTP, noninvasive follicular thyroid neoplasm with papillary-like nuclear features; FTC, follicular thyroid carcinoma; OFVPTC, oncocytic follicular variant papillary thyroid carcinoma; OCA, oncocytic carcinoma; WDT-UMP, well differentiated tumor of uncertain malignant potential; PDTC, poorly differentiated thyroid carcinoma. Global *p* value represents the comparison of the overall distribution of WHO histopathological diagnoses between Bethesda III and Bethesda IV nodules using the Monte Carlo Fisher–Freeman–Halton exact test.

**Table 5:** Aggressive carcinoma.

Aggressiveness	Bethesda III (%)	Bethesda IV (%)	*p* Value	Effect (B IV vs. B III)	95% CI
Non-aggressive	90 (88.2)	86 (85.1)			
Aggressive Tumors	12 (11.8)	15 (14.9)	0.517	Risk difference: 3.1 pp	−6.4 to 12.7 pp
				Odds ratio: 1.31	0.58 to 2.89
*BRAF V600E*	3	2			
*EIF1AX*, *GNAS*, *TERT*	0	1			
*HRAS*	0	4			
*KRAS*	3	0			
*NRAS*	3	2			
*PTEN*	1	0			
*TERT*	0	1			
*THADA*/*IGF2BP3*	0	1			
*PAX8*/*PPARG*	1	1			
*CCDC6*/*RET*	1	0			
*NTRK3*/*ETV6*	0	1			
*CNA*	0	1			
*GEP*	0	1			

Aggressive carcinoma was defined by the presence of gross extrathyroidal extension (ETE), lymphovascular invasion (LVI), lymph node metastasis (LNM), high-risk histological features, or aggressive histological subtypes (including tall cell, columnar, hobnail/micropapillary, and diffuse sclerosing variants [18].

**Table 6:** Capsular, vascular, and widely invasive disease.

Invasiveness	Bethesda III (%)	Bethesda IV (%)	*p* Value	Effect (B IV vs. B III)	95% CI
Minimally Invasive	34 (97.1)	30 (78.9)			
Invasive	1 (2.9)	8 (21.1)	0.029	Risk difference: 18.2 pp	2.8 to 33.7 pp
				Odds ratio: 9.07	1.06 to 38.89
*EIF1AX*	0	1			
*HRAS*, *TERT*	1	0			
*THADA*/*IGF2BP3*	0	1			
*PAX8*/*PPARG*	0	2			
*CNA*	0	4			

Minimally invasive was defined as capsular invasion only, without vascular invasion. Invasive included tumors with focal (<4 foci) or extensive (≥4 foci) vascular invasion, as well as widely invasive carcinomas [19].

## Data Availability

Data supporting the findings of this study can be obtained from the corresponding author upon reasonable request. Public sharing of the data is restricted in accordance with the ethics approval agreement.

## Abbreviations

The following abbreviations are used in this manuscript:

AF	Allele frequency
ATA	American Thyroid Association
AUS	Atypia of undetermined significance
CAD	Canadian dollars
CNA	Copy number alteration
ETE	Extrathyroidal extension
FN	Follicular neoplasm
FTC	Follicular thyroid carcinoma
GEP	Gene expression profile
IEFVPTC	Invasive encapsulated follicular variant papillary thyroid carcinoma
INESSS	Institut national d’excellence en santé et en services sociaux
ITN	Indeterminate thyroid nodule
LNM	Lymph node metastasis
LVI	Lymphovascular invasion
NBNR	Non-BRAF/non-RAS
NGS	Next-generation sequencing
NIFTP	Noninvasive follicular thyroid neoplasm with papillary-like nuclear features
OCA	Oncocytic carcinoma
OFVPTC	Oncocytic follicular variant papillary thyroid carcinoma
OR	Operating room
PDTC	Poorly differentiated thyroid carcinoma
PPV	Positive predictive value
PTC	Papillary thyroid carcinoma
QOL	Quality of life
RAMQ	Régie de l’assurance maladie du Québec
ROM	Risk of malignancy
SNV	Single nucleotide variant
TBSRTC	The Bethesda System for Reporting Thyroid Cytopathology
TI-RADS	Thyroid Imaging Reporting and Data System
USFNA	Ultrasound-guided fine-needle aspiration
WDT-UMP	Well differentiated tumor of uncertain malignant potential
WHO	World Health Organization
